# Altered glucocorticoid reactivity and behavioral phenotype in rx3-/- larval zebrafish

**DOI:** 10.3389/fendo.2023.1187327

**Published:** 2023-07-06

**Authors:** Ulrich Herget, Soojin Ryu, Rodrigo J. De Marco

**Affiliations:** ^1^ Research Group Developmental Genetics of the Nervous System, Max Planck Institute for Medical Research, Heidelberg, Germany; ^2^ Division of Biology and Biological Engineering, California Institute of Technology, Pasadena, CA, United States; ^3^ Living Systems Institute, College of Medicine and Health, University of Exeter, Exeter, United Kingdom; ^4^ School of Biological and Environmental Sciences, Faculty of Science, Liverpool John Moores University, Liverpool, United Kingdom

**Keywords:** pituitary, rx3, stress, cortisol, zebrafish, HPA axis, glucocorticoid

## Abstract

**Introduction:**

The transcription factor rx3 is important for the formation of the pituitary and parts of the hypothalamus. Mutant animals lacking rx3 function have been well characterized in developmental studies, but relatively little is known about their behavioral phenotypes.

**Methods:**

We used cell type staining to reveal differences in stress axis architecture, and performed cortisol measurements and behavior analysis to study both hormonal and behavioral stress responses in rx3 mutants.

**Results and Discussion:**

Consistent with the role of rx3 in hypothalamus and pituitary development, we show a distinct loss of corticotrope cells involved in stress regulation, severe reduction of pituitary innervation by hypothalamic cells, and lack of stress-induced cortisol release in rx3 mutants. Interestingly, despite these deficits, we report that rx3-/- larval zebrafish can still display nominal behavioral responses to both stressful and non-stressful stimuli. However, unlike wildtypes, mutants lacking proper pituitary-interrenal function do not show enhanced behavioral performance under moderate stress level, supporting the view that corticotroph cells are not required for behavioral responses to some types of stressful stimuli but modulate subtle behavioral adjustments under moderate stress.

## Introduction

The hypothalamus-pituitary-adrenal (HPA) axis integrates sensory inputs and generates a hormonal response to support an animal’s struggle against stressors (1, 2). The hypothalamic control center mediating this response is the paraventricular nucleus (PVN) in mammals, or the homologous neurosecretory preoptic area (NPO) in fish (3–5). The pituitary of fish is homologous to the pituitary of other vertebrates, and the homolog of the adrenal gland in fish is known as the interrenal gland (6). The fish version of the HPA axis is therefore termed HPI axis (7–9) and is already functional in larval zebrafish (10, 11). Neuroendocrine cells in the PVN/NPO release corticotropin-releasing-hormone (CRH) and arginine-vasopressin (AVP) in the pituitary, triggering the release of adrenocorticotropic hormone (ACTH) from corticotrope cells, which then acts on steroidogenic cells of the adrenal/interrenal gland, causing the release of glucocorticoids like cortisol as the final stress axis effectors (12). Important PVN/NPO cell types relevant for stress regulation are those producing CRH [primary stress peptide (13)] AVP [known for osmoregulation and vasoconstriction, but also co-acting with CRH (14, 15)] and oxytocin [OXT, mostly known for social functions, but also involved in stress (16)]. OXT has also been implied in promoting the formation of pituitary vascularization (17).

The neurohypophysis in teleosts consists of fiber bundles protruding dorsally into the larger adenohypophysis (17, 18). Adenohypophyseal cells are arranged in distinct subregions [rostral lactotropes and corticotropes; intermediate somatotropes, thyrotropes, gonadotropes; caudal melanotropes and corticotropes (19–21)]. Corticotropes express proopiomelanocortin (POMC), a precursor of ACTH in mammals and fish (22–24). One essential transcription factor for the development of the pituitary and part of the hypothalamus is Rax (mouse) or rx3 (zebrafish), which also is essential for optic cup formation and eye development (25, 26). Loss of Rax/rx3 function appears to prevent proper hypothalamic patterning, since transcription factors and cell type markers including *pomc* and *avp* are not expressed in the hypothalamus of mutants (26–29). *oxt* expression was however reported not to be affected (27) in rx3 mutants. The pituitary fails to develop properly as well, with inhibited formation of the neurohypophysis (30, 31) and malformation of the adenohypophysis that however still produces secretory cells (32). The rx3 mutant fish adenohypophysis has seemingly normal *pomc*-expressing caudal melanotropes, but lacks rostral corticotropes, leading to glucocorticoid deficiency (27).

Corticotroph cells produce several active peptides derived from the cleavage and processing of the precursor gene POMC (33). These POMC-derived peptides can lead to behavioral change when injected into rats (34). Furthermore, ACTH fragments devoid of adrenal function can also prompt behavioral change (35), although mechanisms responsible for rapid behavioral effects of pituitary peptides have yet to be reported. Also, fast glucocorticoid effects on neural activity have been documented in multiple brain areas in mammals (36–38), and behavioral correlates of glucocorticoid injections occurring within minutes after injection have also been reported (39–41). However, despite these advances, the limited accessibility of the hypothalamus, pituitary, and adrenal gland along with the coupled release of brain neuropeptides and peripheral hormones upon stress onset has made it difficult to specify rapid behavioral modulation by the pituitary-adrenal leg of the HPA axis.

Larval zebrafish are particularly suitable for neuroendocrine research since the tissues involved are functionally conserved and optically accessible and behavioral testing can be performed with full control of the environment (11, 42–45). Recent evidence from larval zebrafish highlights the role of the pituitary-interrenal leg of the HPI axis in rapidly modulating adaptive responses to stressors (43). Using optogenetic manipulation of pituitary corticotrophs in combination with novel assays for measuring goal-directed actions in short timescales, we showed that increased corticotroph cell activity upon the onset of stress can enhance cortisol release from steroidogenic cells and allow rapid adjustments of locomotion, stressor avoidance, and stimulus responsiveness (i.e., performance), which aid in coping with stressors (43). We hypothesized that such enhanced performance (43, 46) may be absent in *rx3^-/-^* mutants due to the lack of the rostral *pomc* cluster. Here, we first tested behavioral responses to both stressful and non-stressful stimuli in *rx3^-/-^* mutant larvae and show that they respond to thermal (stressful) and subtle mechanosensory (non-stressful) inputs as wild-type (*rx3^+/+^*) larvae do. Then, we report the absence of stress-induced performance enhancement along with changes in neurosecretory cell projections, neurohypophyseal innervation, and stress hormone release when rx3 function is lost.

## Results

### Larvae lacking rx3 do not show increased levels of circulating cortisol after stressor exposure

**Figure 1:**
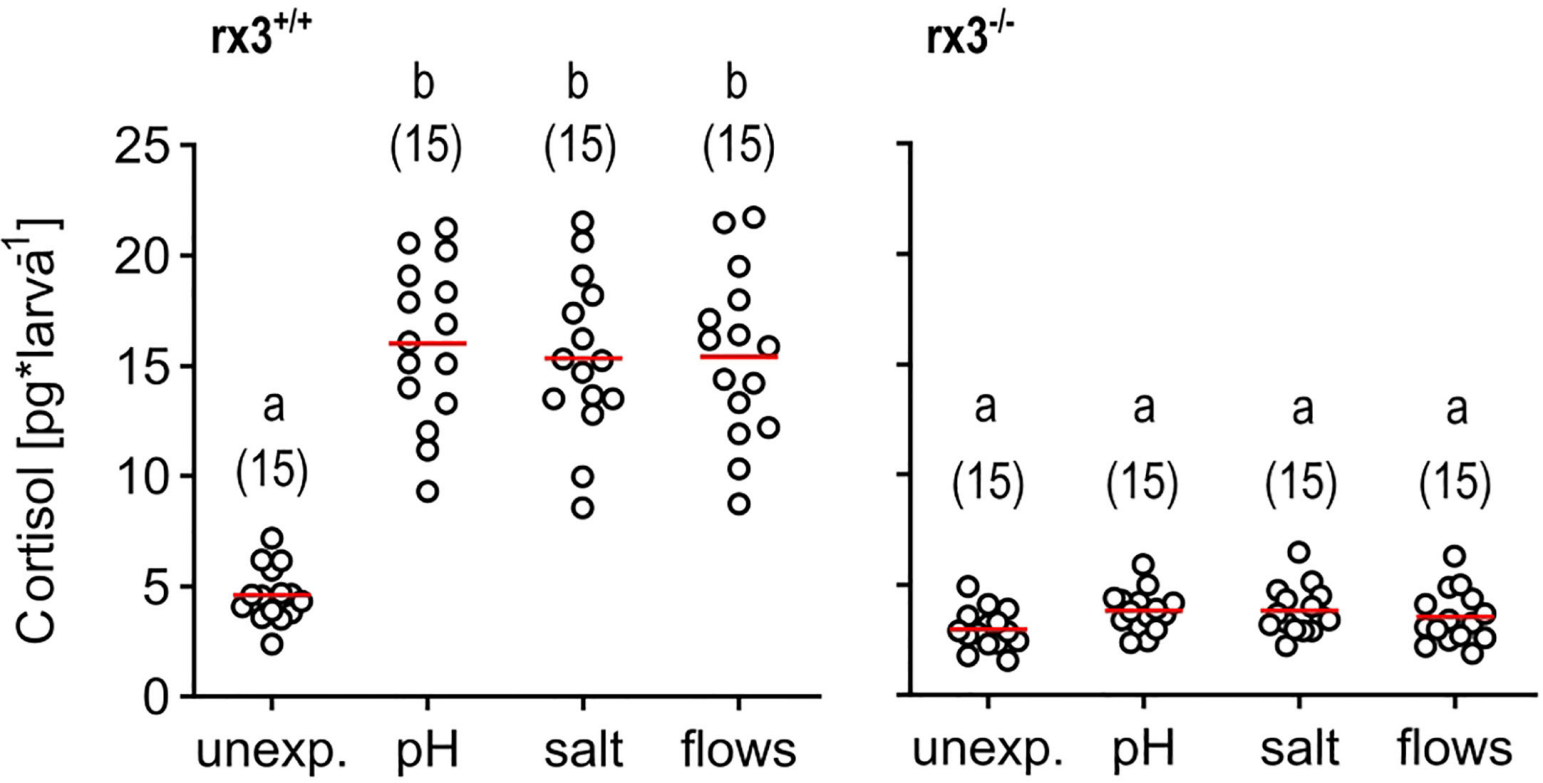
Glucocorticoid reactivity in *rx3^+/+^* and *rx3^-/-^* zebrafish larvae. Whole-body cortisol in *rx3^+/+^* (left) and *rx3^-/-^* (right) larvae (all data points shown, mean in red, sample size in parentheses) after exposure to a pH drop (pH), hyperosmotic medium (salt) or stress-evoking fast water flows (flows). Baseline levels are those of control (unexposed) larvae (unexp.), which were equally handled, omitting stressor exposure. Letters indicate results of Bonferroni’s tests (*p* < 0.001) after one-way ANOVAs (left, *rx3^+/+^*: F(3,59)=42.8, *p* < 0.0001, right, *rx3^-/-^*: F(3,59)=2.7, *p* = 0.1), followed by *post hoc* comparisons.

We deployed three main approaches. Firstly, we exposed wild-type (*rx3^+/+^*) and mutant (*rx3^-/-^*) larvae to a stressor of moderate intensity. To pinpoint effects invariant to stressor identity, we used not just one, but three different stressors: pH drop (pH), hyperosmotic medium (salt), or fast water flows (flows) evoking mechanosensory stress (see also Methods). Each of these stimulations causes cortisol, the final effector of the HPI axis in humans and zebrafish, to increase in a stimulus intensity-dependent manner, as established elsewhere (10, 43, 46–49). To compare the level of HPI axis activation produced by these stimulations, we measured whole-body cortisol directly after stressor exposure. We found that *rx3^-/-^* mutants did not show increased levels of cortisol after stressor exposure, in contrast to wild-type larvae (**Figure 1**), confirming altered HPI axis function upon stress onset in *rx3* ***^-/-^*** mutants.

### HPI axis elements in rx3^+/+^ and rx3^-/-^ larvae

**Figure 2:**
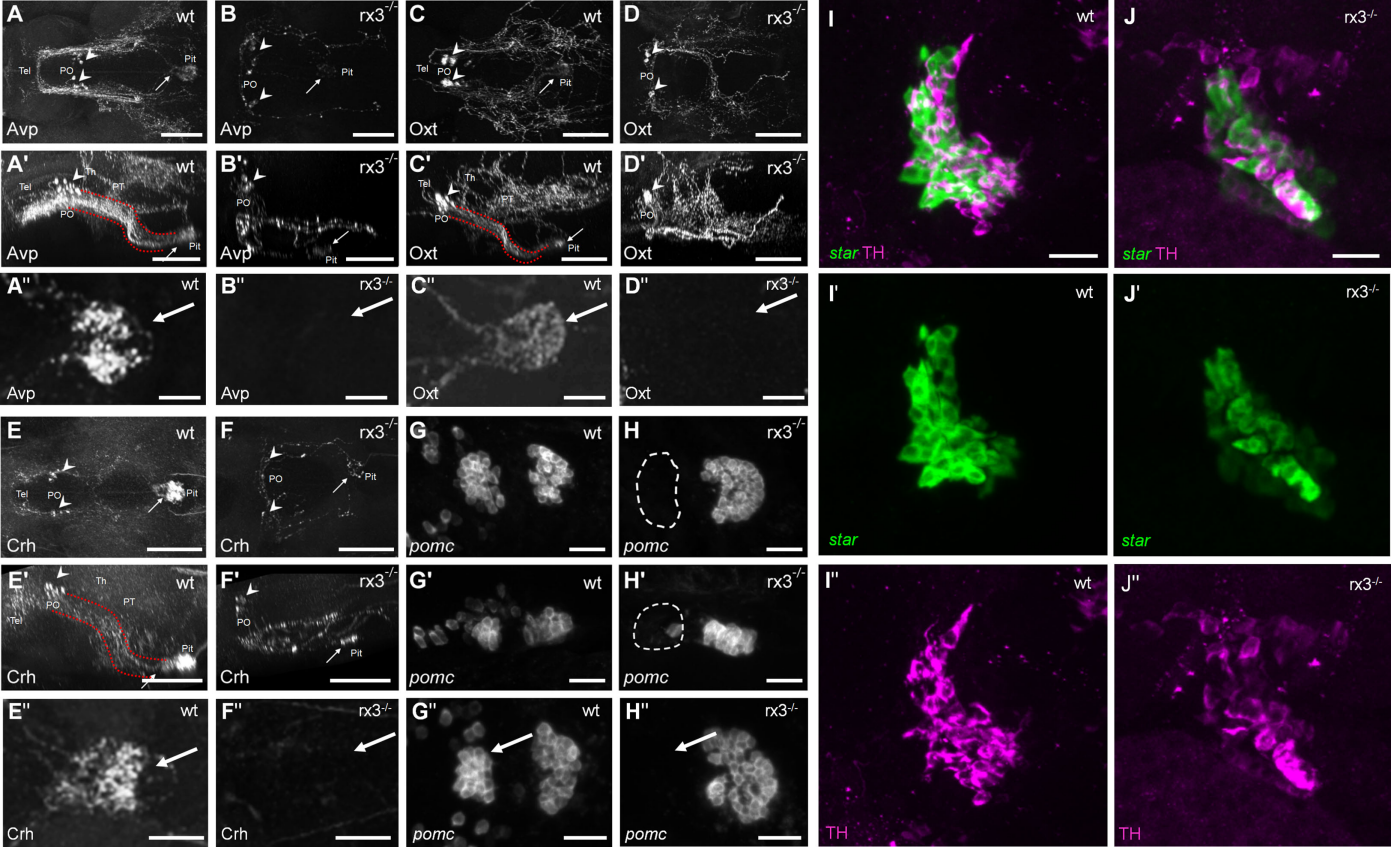
HPI axis elements in *rx3^+/+^* and *rx3^-/-^* zebrafish larvae. **A-F’’**, The hypothalamic part of the HPI axis, illustrated by dorsal **(A, B, C, D, E, F)** and lateral (**A’**, **B’**, **C’**, **D’**, **E’**, **F’**) views of dorsally imaged IHC stainings of three NPO cell types. Cells producing Avp (**A**-**A’**), Oxt (**C**-**C’**), or Crh (**E**-**E’**) form a cluster in the NPO (arrowheads), and many of their fibers innervate the pituitary (arrows) in ***rx3^+/+^*** wild-types (wt) *via* the hypothalamo-hypophyseal tract (red dotted lines). See also dense innervation in dorsal pituitary closeup views of more examples (**A’’**, **C’’**, **E’’**). In *rx3^-/-^* mutant larvae, cells producing Avp (**B**-**B’**), Oxt (**D**-**D’**), or Crh (**F**-**F’**) cluster less densely in the NPO (arrowheads) and their numbers are reduced. Pituitary innervation is present in some of the stainings (e.g., arrows in **B**, **B’**, **F**, **F’**) but not in others (e.g., **D**, **D’**); generally, the projection patterns appear drastically different. See also complete lack of innervation in dorsal pituitary closeup views of more examples (**B’’**, **D’’**, **F’’**). **G**-**H**, The pituitary part of the HPI axis, illustrated by dorsal **(G, H)** and lateral (**G’**, **H’**) images of ISH stainings for *pomc*. Two pituitary clusters are formed by *pomc*-positive cells in ***rx3^+/+^*** wild-types (**G**-**G’**). The rostral cluster is absent, and the caudal cluster appears larger in *rx3^-/-^* mutants (**H**-**H’**). Another example is shown for each case as well (**G’’**, **H’’). I**-**J’**, The interrenal part of the HPI axis, illustrated by combined staining for *star* (**I’**, **J’**, ISH, steroidogenic part) and TH (**I’’**, **J’’,** IHC, chromaffin part). The interrenal gland is intact and both cell types are present in ***rx3^+/+^*** wild-types (**I**-**I’’**) and *rx3^-/-^* mutants (**J**-**J’’**). Abbreviations: Avp, arginine vasopressin; Crh, corticotropic hormone; Oxt, oxytocin; PO, preoptic area; pomc, proopiomelanocortin; Pit, pituitary; PT, posterior tuberculum; Tel, telencephalon; Th, thalamus. Rostral to the left. Scale bars: 100 µm (**A**-**D’**, **E**-**F’**), 25 µm (**A’’**-**D’’**, **E’’**, **F’’**, **G**-**H’’**, **I**, **J**).

Secondly, we examined the expression of specific hypothalamic, pituitary and interrenal markers in *rx3^+/+^* and *rx3^-/-^* mutants (**Figure 2**): corticotropin-releasing hormone (Crh), arginine vasopressin (Avp) and oxytocin (Oxt) in the NPO, POMC in the pituitary (19), and steroidogenic acute regulatory protein (StAR) and tyrosine hydroxylase (TH) in the interrenal gland. Previous work showed that NPO cells in *rx3 ^-/-^* mutant larvae express Oxt or Crh (27). Using specific antibodies against Avp, Oxt and Crh, we confirmed the presence of somata producing these peptides in *rx3 ^-/-^* mutants and found that, compared to *rx3^+/+^* larvae, the cell numbers appeared reduced in larvae lacking rx3 (*rx3^+/+^*: averages of 10 *avp* cells, n=3; 25.25 *oxt* cells, n=4; 8.33 *crh* cells, n=3; *rx3^-/-^*: 5 *avp*, 9 *oxt*, 3.7 *crh* cells, n=3; **Figures 2A-F’**). Immunohistochemical staining (IHC) also revealed alterations in the projection patterns of NPO cells producing Avp, Oxt or Crh in *rx3 ^-/-^* mutants. Fibers of the hypothalamohypophyseal tract connecting the NPO to the pituitary (**Figures 2A’, C’, E’**) were much less organized (**Figure 2B, B’, F, F’**), and pituitary innervation was not always detectable (e.g., **Figure 2B’’, D-D’’, F’’**). Fluorescent *in situ* hybridization (ISH) confirmed the drastic reduction of the rostral *pomc* cluster in the adenohypophysis of *rx3 ^-/-^* mutants (**Figure 2G-H’’**) found previously (27). In addition, the remaining caudal cluster of *pomc* cells appeared to be enlarged in comparison to wildtype larvae (*rx3^+/+^*: averages of 27 rostral and 32.75 caudal *pomc* cells, n=4; *rx3^-/-^*: 2 rostral and 47 caudal *pomc* cells, n=3). Furthermore, analysis of the interrenal gland combining ISH for *star*-positive steroidogenic cells (homologous to the adrenal cortex) and IHC for intermingled TH-positive chromaffin cells (homologous to the adrenal medulla) showed that both cell types are present and appear to be unaffected in *rx3* ***^-/-^*** mutants (**Figure 2I- J’’**). These observations are consistent with the view that altered HPI axis function in *rx3 ^-/-^* mutants emerges from incomplete pituitary innervation and the absence of the rostral *pomc* cluster, and not from analogous morphological alterations of the interrenal gland.

### rx3^-/-^ mutants respond to stressful and non-stressful stimuli, but do not show stress-induced performance enhancement

Thirdly, using established protocols (43, 46, 50), we exposed *rx3^+/+^* vs. *rx3^-/-^* larvae to the above three different stressors of moderate intensity (**Figure 1**, see also **Figures 1A-C** in 46) and compared their post-stress onset performance on distinct innate behaviors driven by either thermal (stressful) or subtle mechanosensory (non-stressful) stimuli. We chose these behaviors because they entail teleonomic actions that can occur in the dark without optical stimulation, thus fitting the eyeless phenotype of the *rx3^-/-^* larvae. Prior to the behavioral tests, we confirmed that *rx3^+/+^* and *rx3^-/-^* larvae had similar levels of basal swimming at 28 °C under infrared illumination (See Methods) (Unpaired two-tailed *t*-test, t_(28)_=0.18, *p* = 0.86; mean distance (mm) swum in 120 s ± S.E.M: *rx3^+/+^*, 484.5 ± 21, N = 15; *rx3^-/-^*, 489.7 ± 20.7, N = 15), in line with previous data (51).

**Figure 3:**
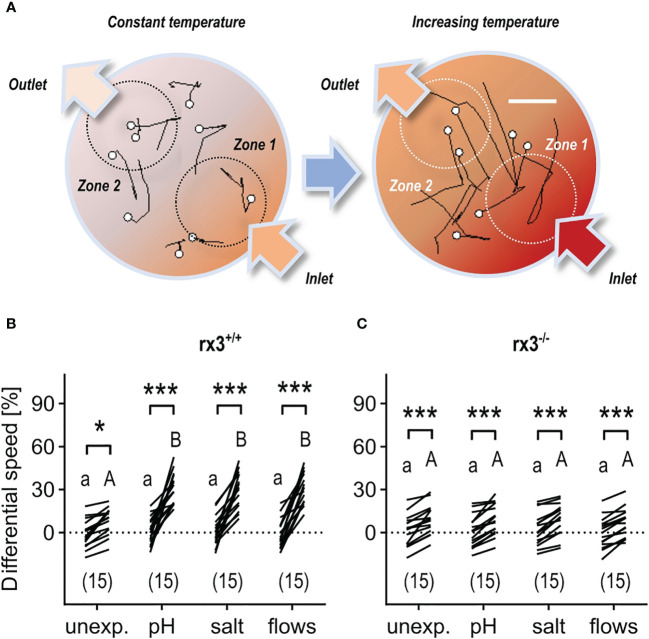
Response to rising temperature in *rx3^+/+^* and *rx3^-/-^* zebrafish larvae under basal and stressed conditions. **(A)** Representative 1 s swim paths from larvae showing increased speed and turns near the inlet (bottom right arrow) as the temperature inside the swimming chamber increases faster in zone 1. White dots indicate start positions. Scale bar, 2.5 mm. Adapted from 43. **(B, C)** Differential speed, i.e., the difference between swim velocity (mm per 40 ms) in zones 1 and 2 (in %), across groups of unexposed (control) and pre-exposed (to either ‘pH’, ‘salt’ or ‘flows’) *rx3^+/+^* **(B)** and *rx3^-/-^* **(C)** larvae before and after the onset of temperature rise (see also Methods). Sample size in parentheses. **(B, C)** Letters and asterisks indicate results of Bonferroni’s tests (*P*<0.001) after two-way repeated measures ANOVAs; **(B)** time factor: F(1,56)=215.2, *P*<0.0001, treatment factor: F(3,56)=7.7, *P*=0.0002, time × treatment factor: F(3,56)=9.2, *P*<0.000; **(C)** time factor: F(1,56)=163.0, *P*<0.0001, treatment factor: F(3,56)=0.2, *P*=0.91, time × treatment factor: F(3,56)=0.3, *P*=0.83.

Larval zebrafish can select best conditions in a thermal-gradient environment (46) and react to rising temperature with fast turns and increased swim velocity (43). Therefore, in a first assay, we examined the relationship between pre-exposure to either ‘pH’, ‘salt’, or ‘flows’, and the actions of individual larvae encountering increasing temperature. For this, we monitored the movements of single larvae swimming in darkness in a cylindrical chamber with opposite inlet/outlet before and after a sharp increase in the temperature of a slowly flowing medium (**Figure 3A**, see also Methods). Due to both heat conduction and the layout of the chamber, the rapidly increasing temperature of the flowing medium caused a substantial temperature difference between the surroundings of the inlet (high temperature, zone 1) and the outlet (low temperature, zone 2). As a result, larvae moving into zone 1 under increasing temperature displayed fast turns and increased swim velocity. To quantify their response to varying temperature, we measured ‘differential speed’ (ΔS) as the difference (in %) between the swim velocity (mm × (40 ms)*^−^* ^1^) in zones 1 and 2 for each larva, or ((swim velocity in zone 1−swim velocity in zone 2)/swim velocity in zone 2) × 100, before (30 s) and after (60–90 s) the onset of the rise in temperature (for details, see Methods and Figures 3C–E in 43). In *rx3^+/+^* larvae, the distribution of ΔS values across groups showed that, under increasing temperature, unexposed (control) subjects swam faster in zone 1, and that larvae pre-exposed to any of the above stressors had equally distributed ΔS values that were, on average, 353.1% higher than those of control larvae (**Figure 3B**). In *rx3^-/-^* larvae, ΔS values also revealed that unexposed subjects swam faster in zone 1 under increasing temperature. However, stressor exposure did not change the distribution of ΔS values across groups of *rx3^-/-^* larvae (**Figure 3C**).

**Figure 4:**
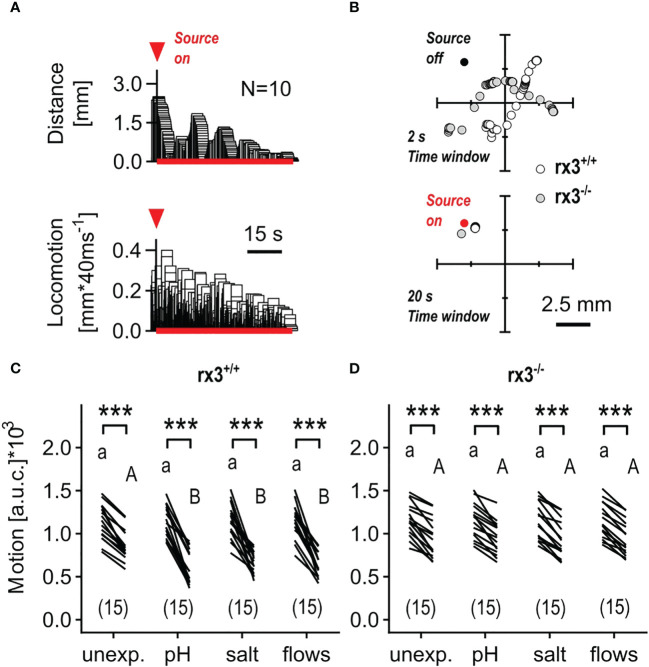
Response to minute water motions in *rx3^+/+^* and *rx3^-/-^* zebrafish larvae under basal and stressed conditions. **(A)** Representative average trace of a wildtype larva’s distance to the stimulus source (top) and swimming activity (bottom) after the onset of mWMs. Red arrow heads in **A** depict the onset of stimulation. (See also **Figure 3** in 50.) **(B)** Representative examples of consecutive x-y coordinates (swim trajectories) measured every 40 msec from *rx3^+/+^* and *rx3^-/-^* larvae without (top) and with (bottom) mWMs. Top, 50 x-y coordinates per larva *without* mWMs (source off) measured over the last *2 s before the onset* of mWMs. Bottom, 500 x-y coordinates per larva *with* mWMs (source on) measured over the last *20 s before the offset* of mWMs. The time window in bottom (20 s) is enlarged 10 times relative to top (2 s) with the sole purpose of highlighting the mWMs-derived lack of locomotion observed typically at the end of a 2 min stimulation period. **(C, D)** Motion level, i.e., the area under the swim velocity-time curve over 120 s, across groups of unexposed and pre-exposed (same groups as in **Figure 3B, C**) *rx3^+/+^* **(C)** and *rx3^-/-^* **(D)** larvae before and during mWMs (see also Methods). Sample size in parentheses. **(C, D)** Letters and asterisks indicate results of Bonferroni’s tests (*P*<0.001) after two-way repeated measures ANOVAs; **(C)** time factor: F(1,56)=359.3, *P*<0.0001, treatment factor: F(3,56)=1.6, *P*=0.20, time × treatment factor: F(3,56)=5.6, *P*=0.002; **(D)** time factor: F(1,56)=317.0, *P*<0.0001, treatment factor: F(3,56)=0.03, *P*=0.99, time × treatment factor: F(3,56)=0.14, *P*=0.94.

Hydrodynamic sensing provides fish with various benefits (52) and larval zebrafish have been shown to respond to subtle, structured (i.e., 1-5 Hz), locally generated water motions (henceforth minute water motions, or mWMs) with reduced locomotion and positive taxis towards the stimulus source (**Figures 4A, B**), a response that is highly sensitive to stimulus features and sensory background (50). In contrast to the above stressful ‘flows’, mWMs are non-stressful (50); they cause structured interactions between a larva’s surroundings and its mechanosensory machinery. Therefore, in a second assay, we compared the response to mWMs of both unexposed (control) and pre-exposed *rx3^+/+^* and *rx3^-/-^* larvae. To quantify a larva’s response to mWMs, we measured integrals of distance swum against time for equal periods before and during stimulation (see also Methods and Figures 3F, G in 43). In *rx3^+/+^* larvae, the distribution of motion values across groups gave responses for pre-exposed subjects that were, on average, 176.9% greater than those of unexposed subjects (**Figure 4C**), in line with previous work (43, 46). In *rx3^-/-^* larvae, by contrast, both unexposed and pre-exposed subjects showed equally distributed values like those of unexposed *rx3^+/+^* larvae (**Figure 4D**). Thus, in *rx3^-/-^* larvae, enhanced performance did not follow the onset of stress. Altogether, the results showed that the (three) different stressors of moderate intensity caused positively correlated changes in cortisol, ΔS and motion values in *rx3^+/+^*, but not in *rx3^-/-^* larvae.

## Discussion

Consistent with the HPI axis functioning as a cascade of one element triggering release from the next, the loss of *pomc* cells in the rostral pituitary (and reduced neuroendocrine cell projections) prevents the increased release of cortisol under stress. This effect resembles impaired cortisol release following ablation of the NPO or the interrenal (42, 44) and supports the assumption that the rostral cluster of *pomc* cells is the part of the pituitary required for HPI axis function.

It was previously reported that *avp* expression is lost in rx3 mutants, and that *oxt* cells seemed to be unaffected (27, 28). Our results show that Crh, Avp, and Oxt are still detectable, but with reduced cell numbers (*avp* -50%, *oxt* -64%, *crh* -56%) and drastically altered projection patterns in rx3 mutants. Formation of the neurohypophysis was shown to be blocked in rx3 mutants (30, 31), which agrees with our observation that Oxt projections do not seem to innervate the pituitary, while Crh and Avp projections to the pituitary are strongly reduced. It is plausible that, like Oxt projections, a neurohypophysiotropic subpopulation of Avp cells no longer projects to the pituitary, since Oxt and Avp are known as the typical magnocellular cell types that project to this part of the pituitary that fails to develop. Avp is however also coexpressed in Crh cells (53–57), and those cells project to the adenohypophysis, which does form at least partially and would explain the sparse and faint innervation we see there. The specific loss of *pomc* expression in the rostral adenohypophyseal corticotrope cluster (rostral *pomc* -93%, caudal *pomc* +44%) was also observed after treatment with the GR agonist dexamethasone (8, 9, 58) and after chronic optogenetic interrenal steroidogenic cell activation (59).

While we focus on the HPI axis, it should be noted that loss of rx3 affects hypothalamic patterning and cell type specification beyond the NPO, pituitary, and interrenal gland. The transcription factors otpa and otpb are expressed in the NPO and both required for NPO cell differentiation (60). Otpb expression is lost in rostral hypothalamic regions in *rx3^-/-^* (29). It is possible that otpa is still expressed and compensates the loss of otpb. Previous results suggested that rx3 is required for proper avp cell differentiation (28), but that oxt and crh are still expressed when rx3 is lost (27). Our results demonstrating the presence of NPO cells do however also show that their neuronal projections are much less organized in *rx3^-/-^* and often fail to reach the pituitary. While both hypothalamic and pituitary development are affected (26), other indirect effects on the stress regulation system, such as impaired axonal pathfinding during development, or changes to forebrain structures beyond the NPO and pituitary, cannot be ruled out.

Apart from experiments on their locomotor reaction to light (61, 62), the behavior of *rx3^-/-^* larvae has remained unexplored until now. Here, we present behavioral data that support the view that *rx3^-/-^* larvae can display complex behavioral schemes like those of wild-type larvae. Further studies focusing on other behavioral reactions/responses to the environment could elucidate how impaired these mutants are, and which types of behavioral motifs remain unaffected despite major alterations in brain development. It is conceivable, for example, that fast reactions involving reflex circuits *via* unaffected brain regions operate just as well as in wild-types, and that more elaborate responses that require suitably developed brain regions like the hypothalamus are significantly hampered in mutants. It is likely that various stress processing pathways (like catecholamine release *via* the sympathetic nervous system) enable mutants to deal with antagonistic environments. Several mutant alleles of rx3 have been described besides the ‘strong’ allele (chkt25327, 66) studied here, including a ‘weak’ allele (chkt25181) that lacks the severe reduction in corticotrope cells seen in the strong rx3 mutants. While still lacking eyes, the weak rx3 mutant larvae retain a wild-type appearance of corticotrope cells and of cells expressing *pomc* in the arcuate nucleus and show normal basal cortisol levels as well as circadian changes in cortisol (27). It would be interesting to examine projection patterns of hypothalamic neurons as well as stressor-derived cortisol reactivity and behavioral task performance in weak rx3 mutants. The strong rx3 mutant’s failure to show enhanced performance upon stress onset (43, 46) however supports the idea that the pituitary-interrenal leg of the HPI axis plays a role in rapidly modulating responses to stressors (43) and opens an opportunity for further analysis of acute stress and behavior under deficient pituitary-adrenal/interrenal interaction. Genetic tools to modify corticotroph and steroidogenic cell activity are being developed (43, 44, 59) and more mutants relevant for steroidogenesis are now available (63, 64). These tools and mutants can be combined with phenotyping, pERK immunostaining, and optogenetics to provide further insights into neuroendocrine and behavioral reactions under altered pituitary/interrenal function.

## Methods

### Zebrafish husbandry and handling

Zebrafish breeding and maintenance were performed under standard conditions (65). Heterozygous *rx3^+/-^* fish (‘strong’ allele chkt25327, 66) were in-crossed and embryos collected in the morning and raised on a 12:12 light/dark cycle in E2 medium at 28 °C. *rx3^+/-^* offspring was screened for homozygous mutants based on morphology (i.e., strong pigmentation and absence of eyes in *rx3^-/-^*), as described elsewhere (66). All experiments were carried out with larvae at 6 days post fertilization (dpf). Tests were performed between 09:00 hours and 18:00 hours, with different experimental groups intermixed throughout the day. Zebrafish experimental procedures were performed according to the guidelines of the German animal welfare law and approved by the local government (Regierungspräsidium Karlsruhe; G-29/12).

### Whole-mount fluorescent *in situ* hybridization, immunohistochemistry, and imaging

Riboprobes for the *steroidogenic acute regulatory protein* (*star*) (9) and *pomc* (19) were synthesized from linearized plasmids following instructions provided with the digoxygenin labeling mix (Roche, #11277073910). Whole-mount fluorescent *in situ* hybridization and immunohistochemistry were performed as described elsewhere (67, 68), using primary rabbit antibodies labeling Avp, Oxt (5), Crh (Advanced Targeting Systems AB-02, RRID : AB_171828), TH [(69), RRID : AB_2631248] and the secondary anti-rabbit antibody Alexa 488 (invitrogen). For imaging, specimens were cleared in 80% glycerol (Gerbu) in PBS for 1 h. Confocal stacks were recorded using a Leica SP5 confocal microscope with a Nikon 20x glycerol objective. Each channel was recorded sequentially to reduce interfering signals from overlapping emission spectra. Zoom, dimensions, gain, offset, average, and speed were adjusted for each stack to obtain the optimal image quality of the desired volume. Stacks were evaluated using Amira 5.4 (Thermo Fisher, SCR_007353) to create maximum intensity projections and rotated voxel views. They were spatially restricted to the volume of interest, excluding signals from planes in front or behind. Brightness and contrast were adjusted for each channel, and somata were counted by eye.

### Stressors

Groups of thirty larvae in 30 mm Petri dishes were exposed to three different stress protocols of moderate intensity using known protocols (11, 43, 46, 47, 70, 71), each based on one of the following stimuli: HCl (pH drop, ‘pH’), NaCl (hyperosmotic medium, ‘salt’), or fast water flows preventing normal swimming (‘flows’). Larvae were then used for cortisol measurement or transferred to a custom-made swimming chamber for behavioral testing. *pH*: larvae were incubated for 3 min in either steady-state E2 medium (unexposed) or E2 + hydrochloric acid (Merck, #109063) (pH = 4) at 28 °C under white-light illumination. They were then washed three times with E2 medium and kept in a small container for cortisol detection (6 min later) or transferred to the swimming chamber for behavioral testing (12 min later). The wash and transfer period lasted 3 min (± 10 s) and was performed at room temperature. *Salt*: larvae were incubated for 10 min in either steady state E2 medium (unexposed) or E2 + NaCl (Merck, #106404) (NaCl concentration = 25 mM) at 28°C under white light illumination. They were also washed three times with E2 medium and kept in a small container for immediate cortisol detection or transferred to the test chamber (5 min later). The wash and transfer period also took 3 min (± 10 s) and was performed at room temperature. *Flows*: larvae were presented with stress-evoking fast water flows (71) caused by the rapid lateral displacements of a silica bar (Polymicro Technologies, AZ, 360 µm OD, Optronis GmbH; Kehl, Germany) fixed to a multilayer piezo bender actuator (PICMA^®^ PL140.10, Physik Instrumente (PI) GmbH and Co. KG; Karlsruhe, Germany). The actuator had an operating voltage of 0–60V, a maximum displacement of ±1000 µm, and an unloaded resonant frequency of 160 Hz. The bender was connected to a dual-piezo-amplifier (maximum voltage: 10 V), a pulse generator and a TTL control box (USB-IO box, Noldus Information Technology, Wageningen, The Netherlands) allowing for computer control. The tip of the silica bar was submerged (2 mm) at the centre of a 30 mm Petri dish, half filled (1.8 ml) with E2 medium (orientation relative to water surface: 90°). The voltage applied to the bender (V_act_) determined the speed of the capillary’s lateral displacements, or stimulus strength (in % relative to maximum voltage). Groups of 30 larvae were exposed to 6 stimulation units delivered with an inter-stimulation-interval of 250 ms. Each unit consisted of 99 repetitions of 40 ms lateral displacements. We used a V_act_ of 3. Stimulations were carried out under white illumination at 28°C. After stimulation, larvae were kept in Petri dishes for cortisol measurement (9.5 min later), or transferred to the swimming chamber for behavioral testing, where they remained without perturbation for 10 min before recordings.

### Independent sampling

Cortisol and behavioral measurements were made on different groups of equally treated larvae and therefore constitute fully independent samples. For the behavioral measurements, each replicate involved a single larva. These individual measurements were made on larvae that had also been kept in wells containing a total of thirty larvae per well. The number of single larvae thus matched the number of independent wells. In this manner, the density of larvae per well during stressor exposure remained a constant factor for both the cortisol and behavioral measurements. For each cortisol measurement, all thirty larvae in a well were used, whereas each behavioral measurement involved only one larva - the remaining twenty-nine larvae in the well were used elsewhere. Each replication was fully independent from the others thus avoiding pseudo-replication.

### Whole-body cortisol

Cortisol extraction and detection were carried out using a customized ELISA protocol, as described elsewhere (70). Each replicate consisted of a well with 30 larvae. The groups of thirty larvae were immobilized in ice water after being exposed to either ‘pH’, ‘salt’, or ‘flows’, as described above. Unexposed larvae (control samples) were collected after equal handling, omitting stressor exposure. Samples were then frozen in an ethanol/dry-ice bath and stored at −20 °C for subsequent extraction.

### Test overview

Behavioral experiments using increasing temperature of the flowing medium or minute water motions were conducted under infrared illumination delivered through a custom-made array of infrared-LEDs mounted inside a light-proof enclosure. The complete behavioral setup was placed on a vibration-free platform (Newport Corp, Irvine, CA, USA). Larvae were imaged at 25 frames s*^−^* ^1^ (camera: ICD-49E B/W, Ikegami Tsushinki Co, Ltd, Japan) with a lens (TV Lens, Computer VARI FOCAL H3Z4512 CS-IR, CBC; Commak, NY, USA) positioned above a cylindrical custom-made swimming chamber. The swimming chamber (internal diameter: 10 mm, height: 10 mm) had a transparent bottom and two opposite overtures, inlet and outlet (width: 2.5 mm, height: 400 μm; (see also Figure S7B, C in 43), allowing E2 medium to constantly flow at 200 μl min*^−^* ^1^ by means of a peristaltic pump (IPC Ismatec, IDEX Health and Science GmbH, Wertheim, Germany). The chamber also had two cylindrical side channels (internal diameter: 400 μm) opposite to each other opening 200 μm above the transparent glass bottom, with their longest axis oriented at an angle of 30° relative to horizontal. One such channel held a thermocouple (TS200, npi electronics GmbH, Tamm, Germany) monitoring the temperature inside the chamber and providing feedback to a control system (PTC 20, npi electronics GmbH; Exos-2 V2 liquid cooling system, Koolance, Auburn, WA, USA) that either kept the flowing medium at 28 °C ( ± 0.1 °C) or increased its temperature rapidly in a highly controlled manner (see below). The second side channel allowed passage of the end of a rigid silica capillary tube, or stimulus source (outer diameter: 350 μm, full length: 25 mm, Polymicro Technologies), submerged ∼400 μm into the chamber’s inner medium (depth: 5 mm). The opposite end of the capillary tube was fixed to a multilayer bender actuator (PICMA PL140.10, Physik Instrumente (PI) GmbH+Co. KG, Karlsruhe, Germany) with an operating voltage of 0–60 V, a maximum displacement of ±1,000 μm and an unloaded resonant frequency of 160 Hz. The bender, coupled to a pulse generator, a dual piezo amplifier and a TTL control system, produced unidirectional lateral displacements (of 50 μm and controllable speed) of the capillary’s submerged end, creating minute water motions (mWMs) within the chamber. The input voltage applied to the actuator (0.5 V) determined the speed of the capillary’s lateral displacements (see also 50). EthoVision XT 7 software (Noldus Information Technology, SCR_000441) was used to monitor the movements of individually swimming larvae. Prior to the test, each larva was given an initial time of ten minutes to adapt to the chamber’s conditions. Experiments were conducted at 28 ± 1°C, unless otherwise stated. A thermocouple (npi electronics GmbH; Tamm, Germany) connected to a temperature control system (PTC 20, npi electronics GmbH; Tamm, Germany; Exos-2 V2 liquid cooling system, Koolance; Auburn, WA, USA) monitored the temperature inside the swimming chamber. All the experiments were performed in a blind fashion as to group identity. Control animals for each group were handled in the same fashion but omitting stressor presentation.

### Thermal input

Single larvae were video recorded for 240 s with the temperature of the flowing medium kept at 28 °C ( ± 0.1 °C). The input of the temperature control system was then stepped up by 10 °C, causing the temperature of the flowing medium to reach 34 °C after 120 s. The rising temperature produced a temperature difference between the area near the inlet (zones 1, high temperature) and the area near the outlet (zone 2, low temperature). Such temperature difference reached its maximum 60–90 s after the onset of temperature rise, a time window used to measure ‘differential speed’. See also Figures 3C, D in 43.

### Minute water motions

Single larvae in the swimming chamber were video recorded for 120 s under infrared light and constant temperature. They were presented with mWMs caused by 1 ms lateral displacements of the silica capillary tube delivered at 1 Hz (input voltage: 0.5 V) for 120 s, as described above and elsewhere (50). Motion before and during stimulation was calculated using the integrals of motion over 120 s.

### Statistics

All data are shown as boxplots (**Figure 1**, median and whiskers: min to max) or as single measurement points (**Figures 3**, **4**). We used a random experimental design and ANOVAs for multiple group comparisons (followed by Bonferroni’s *post hoc* tests). Normality was tested using Kolmogorov–Smirnov, Shapiro–Wilk and D’Agostino tests. Analyses were made with MS-Excel (Microsoft Corp; Redmond, WA, USA, SCR_016137) and Prism 9 (Graphpad Software Inc, San Diego, CA, USA, SCR_002798).

## Data availability statement

The datasets presented in this study can be found in online repositories. The names of the repository/repositories and accession number(s) can be found in the article/**Supplementary Material**.

## Ethics statement

The animal study was reviewed and approved by Regierungspräsidium Karlsruhe; G-29/12.

## Author contributions

RM and SR conceived experiments, RM designed experiments, RM and UH performed experiments, analyzed results, and wrote the paper. All authors contributed to the article and approved the submitted version.
